# The IgA Isotype of Anti-β2 Glycoprotein I Antibodies Recognizes Epitopes in Domains 3, 4, and 5 That Are Located in a Lateral Zone of the Molecule (L-Shaped)

**DOI:** 10.3389/fimmu.2019.01031

**Published:** 2019-05-07

**Authors:** Manuel Serrano, Jose Angel Martinez-Flores, Gary L. Norman, Laura Naranjo, Jose Maria Morales, Antonio Serrano

**Affiliations:** ^1^Healthcare Research Institute of Hospital 12 de Octubre, Madrid, Spain; ^2^INOVA Diagnostics, San Diego, CA, United States

**Keywords:** antiphospholipid antibodies, antiphospholipid syndrome, kidney transplant, graft thrombosis, epitope mapping, peptide arrays, B2GP1

## Abstract

**Background:** Antiphospholipid syndrome (APS) is characterized by thrombosis and/or pregnancy morbidity with presence of anti-phospholipid antibodies (aPL). The APS classification criteria only consider the aPL of IgG/IgM isotype, however testing of aPL of IgA isotype is recommended when APS is suspected and consensus aPL are negative. IgA anti-βeta-2 glycoprotein-I (B2GP1) has been clearly related with occurrence of thrombotic events. Antibodies anti-B2GP1 of IgG/M isotypes recognize an epitope in Domain 1 (R39-G43), the epitopes that recognize IgA anti-B2GP1 antibodies are not well-identified.

**Aim:** To determine the zones of B2GP1 recognized by antibodies of IgA isotype from patients with APS symptomatology and positive for IgA anti-B2GP1.

**Methods:** IgA antibodies to Domain-1(D1) and Domain-4/5(D4/5) of B2GP1 (ELISA) and epitope mapping on oligopeptide arrays of B2GP1 were evaluated in sera from a group of 93 patients with at least one thrombotic and with isolated positivity for IgA anti-B2GP1 antibodies (negative for other aPL).

**Results:** A total of 47 patients (50.5%) were positive for anti-D4/5 and 23(25%) were positive for anti-D1. When peptide arrays were analyzed, three zones of B2GP1 reactivity were identified for more than 50% of patients. The center of these zones corresponds to amino acids 140(D3), 204(D4), and 264(D5). The peptides recognized on D3 and D4 contain amino acid sequences sharing high homology with proteins of microorganism that were previously related with a possible APS infectious etiology. In the three-dimensional structure of B2GP1, the three peptides, as the R39-G43 epitope, are located on the right side of the molecule (L-shape). The left side (J-shape) does not bind the antibodies.

**Conclusions:** Patients with thrombotic APS clinical-criteria, and isolated IgA anti-B2GP1 positivity appear to preferentially bind, not to the D1 or D4/5 domains of B2GP1, but rather to three sites in D3, D4, and D5. The sites on D3 and D4 were previously described as the target identified by human monoclonal antibodies derived from patients that were capable of inducing APS in animal models. The localization of these epitopes opens a new route to explore to increase understanding of the patholophysiology of the APS and to propose new alternatives and therapeutic targets.

## Introduction

Antiphospholipid syndrome (APS) is an autoimmune vascular disorder characterized by recurrent thrombosis and gestational morbidity in carriers of antiphospholipid autoantibodies (aPL) (1).The most common form of the disease is the Primary antiphospholipid syndrome (P-APS) (2). There are two other forms of APS, one is that associated with systemic autoimmune diseases (SAD-APS), mainly systemic lupus erythematosus (SLE) and the other catastrophic APS (3, 4).

The main antigenic target for aPL is Beta 2 Glycoprotein I (B2GP1), a plasma protein that can circulate freely in the blood or that is bound to lipoproteins or phospholipids, like the cardiolipin (5).

B2GP1 is composed of 5 short consensus repeat domains (“sushi” domains). There are several different conformations for B2GP1, the most important of which are the circular plasma conformation and an open conformation which resembles a fish-hook or “*hockey* stick” (6, 7).

The classification criteria of APS established by an International consensus statement in 2006 are based on the simultaneous presence of at least one clinical and one laboratory criterion. The laboratory criteria include positivity of any lupus anticoagulant, anticardiolipin antibodies (aCL) and/or of anti-B2GP1, persistently positive and at medium to high titer. Only antibodies of IgM and IgG Isotypes were included in classification criteria. IgA anti-BGP1 antibody positivity was not included due to lack of sufficient supporting evidence at that time (1).

The question of whether IgA anti-B_2_GPI may have diagnostic value for APS was subsequently addressed by the “non-criteria” antiphospholipid task force during the 13th International Congress on Antiphospholipid Antibodies held in April 2010 in Galveston, Texas. The task force concluded that the IgA anti-β2GP1 antibodies should be tested in patients with clinical signs and symptoms of APS, particularly when other antiphospholipid tests are negative (8). Importantly, IgA anti-β2GP1 antibodies are included as serologic markers of SLE in the revised classification criteria for SLE (9).

Over the past several years interest in the significance of anti-B2GP1 antibodies of the IgA isotype has been growing. IgA anti-B2GP1 has been shown to be strongly associated with DVT and stroke (10) and the thrombogenic effects of IgA anti-β2GP1 antibodies *in vitro* and *in vivo* animal models has been shown (11). Along this line our group shown that the presence of IgA anti-B2GP1 was strongly associated with thrombosis in various situations including: thrombosis in hemodialyzed patient (12), graft-thrombosis after kidney transplantation (13), and thrombosis and mortality after heart transplantation (14).

It has been described that anti-B2GP1 antibodies of IgG/M isotypes recognize epitopes on all 5 domains of B2GP1 (15, 16) with a conformational epitope in Domain 1 (R39-G43) being the immunodominant target (17). Although it is known that 50% of IgA anti-B2GP1 positive patients have specificity to D4/5, the epitopes that are targeted by IgA anti-B2GP1 antibodies are not well-identified.

The aim of the study is to define the zones of B2GP1 recognized by anti-B2GP1 antibodies of IgA isotype and to determine if these epitopes are similar or different from those described for IgG and IgM anti-B2GP1 isotypes.

## Methods

### Patients and Serum Samples

The sera of patients were obtained from the APS Serum Collection of the Hospital 12 de Octubre Immunology Department (period 2011–2014). Initially it was planned to study 100 patients with thrombotic antecedents: 50 with Stage 4 of Chronic kidney disease (CKD) on the waiting list for renal transplant and 50 with normal renal function (NKF), plus 25 asymptomatic carriers. For reasons of peptide array availability, the number of sera studied was reduced to 93 patients with thrombotic antecedents and 18 asymptomatic controls. The selection criteria for patient group were that they had at least one thrombotic event consistent with clinical APS classification criteria and that they were only positive for IgA anti-β2GP1 (negative for other aPL). All patients were IgA anti-B2GP1 “true positive” (positivity confirmed in at least two samples separated by more than 6 months). Fifty patients were selected from among those who suffered CKD and 43 were selected from among the NKF patients. Nine of the 93 patients (all in the NKF group) had concomitant autoimmune diseases (patient selection and disposition, Supplementary Figure 1).

### CKD Subgroup

Patients in the CKD group had vascular thrombosis during their time on dialysis or in early post-transplant period. Twenty patients were selected because they had thrombotic episodes during the time period when they were undergoing dialysis (regardless of whether they also had thrombosis in the post-transplant). The other 30 were selected because they had thrombosis in the first 3 months of the post-transplant period (regardless of whether they also had thrombosis in the pre-transplant period). Fourteen of these patients lost their graft due to graft-thrombosis in the first 3 months after transplant. All the sera were obtained at the time of the dialysis (before the transplant).

Many of these patients also had other thrombotic events in addition to those used to select them, so that the total number of thrombotic events is greater than the number of patients (Table 1).

**Table 1:** Description of the 79 peptides used in the peptide-array.

**Peptide number**	**Sequence**	**Position**	**Domain**	**Peptide number**	**Sequence**	**Position**	**Domain**
1	GRTCPKPDDLPFSTV	1–15	1	41	MFGNDTITCTTHGNW	161–175	3
2	PKPDDLPFSTVVPLK	5–19	1	42	DTITCTTHGNWTKLP	165–179	3
3	DLPFSTVVPLKTFYE	9–23	1	43	CTTHGNWTKLPECRE	169–183	3
4	STVVPLKTFYEPGEE	13–27	1	44	GNWTKLPECREVKCP	173–187	3–4
5	PLKTFYEPGEEITYS	17–31	1	45	KLPECREVKCPFPSR	177–191	3–4
6	FYEPGEEITYSCKPG	21–35	1	46	CREVKCPFPSRPDNG	181–195	3–4
7	GEEITYSCKPGYVSR	25–39	1	47	KCPFPSRPDNGFVNY	185–199	4
8	TYSCKPGYVSRGGMR	29–43	1	48	PSRPDNGFVNYPAKP	189–203	4
9	KPGYVSRGGMRKFIC	33–47	1	49	DNGFVNYPAKPTLYY	193–207	4
10	VSRGGMRKFICPLTG	37–51	1	50	VNYPAKPTLYYKDKA	197–211	4
11	GMRKFICPLTGLWPI	41–55	1	51	AKPTLYYKDKATFGC	201–215	4
12	FICPLTGLWPINTLK	45–59	1	52	LYYKDKATFGCHDGY	205–219	4
13	LTGLWPINTLKCTPR	49–63	1–2	53	DKATFGCHDGYSLDG	209–223	4
14	WPINTLKCTPRVCPF	53–67	1–2	54	FGCHDGYSLDGPEEI	213–227	4
15	TLKCTPRVCPFAGIL	57–71	1–2	55	DGYSLDGPEEIECTK	217–231	4
16	TPRVCPFAGILENGA	61–75	1–2	56	LDGPEEIECTKLGNW	221–235	4
17	CPFAGILENGAVRYT	65–79	2	57	EEIECTKLGNWSAMP	225–239	4
18	GILENGAVRYTTFEY	69–83	2	58	CTKLGNWSAMPSCKA	229–243	4
19	NGAVRYTTFEYPNTI	73–87	2	59	GNWSAMPSCKASCKV	233–247	4–5
20	RYTTFEYPNTISFSC	77–91	2	60	AMPSCKASCKVPVKK	237–251	4–5
21	FEYPNTISFSCNTGF	81–95	2	61	CKASCKVPVKKATVV	241–255	4–5
22	NTISFSCNTGFYLNG	85–99	2	62	CKVPVKKATVVYQGE	245–259	5
23	FSCNTGFYLNGADSA	89–103	2	63	VKKATVVYQGERVKI	249–263	5
24	TGFYLNGADSAKCTE	93–107	2	64	TVVYQGERVKIQEKF	253–267	5
25	LNGADSAKCTEEGKW	97–111	2	65	QGERVKIQEKFKNGM	257–271	5
26	DSAKCTEEGKWSPEL	101–115	2	66	VKIQEKFKNGMLHGD	261–275	5
27	CTEEGKWSPELPVCA	105–119	2	67	EKFKNGMLHGDKVSF	265–279	5
28	GKWSPELPVCAPIIC	109–123	2–3	68	NGMLHGDKVSFFCKN	269–283	5
29	PELPVCAPIICPPPS	113–127	2–3	69	HGDKVSFFCKNKEKK	273–287	5
30	VCAPIICPPPSIPTF	117–131	2–3	70	VSFFCKNKEKKCSYT	277–291	5
31	IICPPPSIPTFATLR	121–135	3	71	CKNKEKKCSYTEDAQ	281–295	5
32	PPSIPTFATLRVYKP	125–139	3	72	EKKCSYTEDAQCIDG	285–299	5
33	PTFATLRVYKPSAGN	129–143	3	73	SYTEDAQCIDGTIEV	289–303	5
34	TLRVYKPSAGNNSLY	133–147	3	74	DAQCIDGTIEVPKCF	293–307	5
35	YKPSAGNNSLYRDTA	137–151	3	75	IDGTIEVPKCFKEHS	297–311	5
36	AGNNSLYRDTAVFEC	141–155	3	76	IEVPKCFKEHSSLAF	301–315	5
37	SLYRDTAVFECLPQH	145–159	3	77	KCFKEHSSLAFWKTD	305–319	5
38	DTAVFECLPQHAMFG	149–163	3	78	EHSSLAFWKTDASDV	309–323	5
39	FECLPQHAMFGNDTI	153–167	3	79	SLAFWKTDASDVKPC	312–326	5
40	PQHAMFGNDTITCTT	157–171	3				

*The text of the sequence is marked in one color for each domain. Domain 1, green; Domain 2, blue; Domain 3, red; Domain 4, black; Domain 5, purple*.

In addition, a reference group of 18 carriers of IgA anti-β2GP1 (negative for other aPL) without any history of vascular events or gestational morbidity and with preserved renal function was analyzed. Of these, 66% were women. Mean age was 61 ± 4 years and 6 (33%) suffered from systemic autoimmune diseases.

### Ethical Issues

The study was approved by the Hospital 12 de Octubre Ethics Committee for Clinical Research (Reference Numbers CEIC PI13/405, CEIC 14/354, and CEIC 15/008).

The patients were not asked to sign an informed consent because the legislation does not require it for observational studies without intervention in which genetic material is not used. To assure the anonymity of the data, including both sera (blood drawn) and the related clinical data, a blind code was assigned to each patient.

### Antiphospholipid Antibodies

Anti-B2GP1 and anticardiolipin autoantibodies of IgA isotype were quantified by enzyme-linked immunosorbent assays (ELISA) using IgA anti-B2GP1 and IgA anticardiolipin QUANTA Lite® (INOVA Diagnostics Inc., San Diego, CA, USA). Antibody levels higher than 20 Units were considered positive (99th percentile of a healthy population in our hospital, *N* = 321) (18). Levels of anti-cardiolipin and anti-B2GP1 of IgG and IgM isotypes were evaluated using BioPLex 2200 multiplex immunoassay system (Bio-Rad, Hercules CA, USA). Antibody levels higher than 18-GPL/mL (aCL IgG), 18 MPL/mL (aCL IgM), and 18 U/mL (anti-B2GP1, IgG/IgM) were considered positive.

The autoantibodies anti-Domain 1 (D1) and Anti-Domain 4/5 (D4/5) of B2GP1 were evaluated with ELISA tests from Inova as previously described (19), changing the secondary antibody to peroxidase-conjugated anti-human IgA (Inova). The control sera available in the kit were used as reference to convert the OD values to UA.

The 99th percentile values of anti-D1 and anti-D4/5 were obtained by using a healthy population in our hospital (blood donors, *N* = 168). Anti-D1 levels higher than 23.8 U and Anti-D4/5 higher than 22 U were considered positive.

We have no data about the presence of lupus anticoagulant (LA) in most patients in the study. LA is not routinely evaluated in patients with a first thrombotic event in our hospital. Following the protocols established in the hospital, LA is only evaluated in patients with recurrent thrombosis and in situations that the hematologist considers appropriate due to the clinical characteristics of each patient.

### Oligopeptide Microarrays

Peptide arrays (CelluSpots) containing 79 peptides of 15-aa derived from the amino acid sequence of B2GP1 (NCBI Reference Sequence: NP_000033.2, Table-fig 2) were purchased from Intavis AG, Cologne, Germany.

The design of the peptides was done with the objective that the antibodies of the patients could recognize any linear B2GP1 peptide with a length of up to 11 amino acids. Peptide 1 contains the first 15 aa. Peptides (from 2 to 79) were “bled” 4 positions in order to maintain an overlap of 11 aa with the previous one. Number, sequence and position of each peptide are described in Table 1.

To identify IgA-binding epitopes on B2GP1, the peptide arrays were first blocked for 3 h on a horizontal shaker at room temperature (RT) with blocking buffer: TBS-T (Tris-buffered saline, 50 mM, pH 7.4, Tween, 2.5%) with 5% skimmed milk powder. After washing for 10 min with TBS-T, patient sera were placed in a dilution of 1:100 in blocking buffer according to previous optimizations of patient serum and secondary antibody concentrations. Serum incubation was performed overnight at 4°C on a horizontal shaker. The slides were washed 3 × for 10 min with TBST on a horizontal shaker at room temperature (RT) and then anti-human-IgA conjugated to alkaline phosphatase (Mabtech AB, Nacka Strand, Sweden) was placed at a dilution of 1:5,000 in blocking buffer and incubated for 2 h at RT.

After washing 5 × for 10 min., detection was performed using NBT/BCIP Color Substrate Solution (0.4 mg/ml NBT; 0.19 mg/ml BCIP; 50 mM MgSO_4_100 mM Tris buffer, pH 9.5;) was prepared from NBT/BCIP Ready-to-Use Tablets (Sigma-Aldrich, St.Louis, Mo, USA), 30 min at room temperature.

Substrate solution was removed and slides were washed 3 × for 10 min. with TBST. The slides were rinsed to eliminate possible remains of salt by a rapid immersion in distilled water and dried at RT.

### Microarray Data Analysis

Images (gray scale, 16 bits) from slides were read-out using an image scanner and quantitation of the signals were quantified by image analysis. The image segmentation for the identification of the spots was made by the “fixed circle” method. The alkaline phosphatase activity of the spots was determined by image analysis using UTHSCSA ImageTool, version 3.0 (University of Texas Health Science Center, San Antonio, TX, USA). Signals from peptides were quantified measuring the Integrated Optical Density (IOD) of the area corresponding to the spot of each peptide.

A positive signal was defined as a positive IOD value after subtraction of background: the mean IOD of the five negative spots (without any peptide) plus two-fold standard deviation derived from 15 random peptides on each microarray slide. This involves a probability of >95% of positivity for a positive signal (20).

Peptides of high antigenicity were defined as those that were recognized by 50% or more of the patients. When several contiguous peptides were recognized by more than 50% of the patients, only the peptide that had the greatest number of recognitions by the patients was considered to have high antigenicity.

### Visualization of 3D Structure of B2GP1

Jmol (21), an open-source Java viewer for chemical structures in 3D (http://www.jmol.org), was used to visualize and analyze the tertiary structure of B2GP1. The data of crystal structure of human B2GP1 were obtained from Protein Data Bank (doi: 10.2210/pdb1C1Z/pdb).

### Statistical Analysis

Association between qualitative variables was determined with the Pearson χ2 test (or Fisher exact test, when appropriate). Mann-Whitney test was used for comparisons in scaled variables with 2 categories. Pearson's correlation coefficient was used to evaluate the association between two continuous variables; the size of Correlation Coefficient was interpreted following the Hinkle's Rule of Thumb (22). Data were processed using Medcalc for Windows version 17.9 (Medcalc Software, Ostend, Belgium). Probabilities <0.05 were considered significant.

## Results

The mean levels of IgA anti-B2GP1 autoantibodies in the 93 patients were 86.7 ± 6.4 U/mL (mean ± standard error). The median was 60.8. Interquartile range (IQR) was 40.1–121.

The values of IgA anti-B2GP1 in the transplanted patients were slightly higher (Median 83.2; IQR: 52–136) than in the patients with renal function (median 49.4; IQR: 33.1–92.3; *p* = 0.009). The mean age of the patients was 57.8 ± 1.7 years (median 59.5; IQR: 43–72). The sex ratio was very balanced: 53% women.

No significant differences were observed in the sex ratio (*p* = 0.715), age (*p* = 0.428) between CKD and NKF groups, except a greater prevalence of pulmonary embolism in NKF patients. Graft loss due to thrombosis and thrombotic events in dialysis vascular access obviously only occurred in the CKD group (Table 2).

**Table 2:** Description of APS events in the 93 patients studied.

**APS event**	**Global**	**Chronic kidney disease patients (N50)**	**Normal kidney function (*N* = 43)**	***P*-value**
Deep venous thrombosis	58 (32.3%)	31 (62%)	27 (62.8%)	0.938
Arterial thrombosis	15 (16.1%)	9 (18%)	6 (14%)	0.597
Pulmonary embolism	16 (17.2%)	1 (2%)	15 (34.9%)	<0.001
Stroke	6 (6.5%)	1 (2%)	5 (11.6%)	0.092
Myocardial infarction	11 (11.8%)	5 (10%)	6 (14%)	0.556
Graft Thrombosis (kidney transplanted patients)	14 (15%)	14 (28%)	–	–
Vascular access thrombosis (dialyzed patients)	22 (23.7%)	22 (44%)	–	–

### Antibodies Against D1 and D4/5

**Figure 1:**
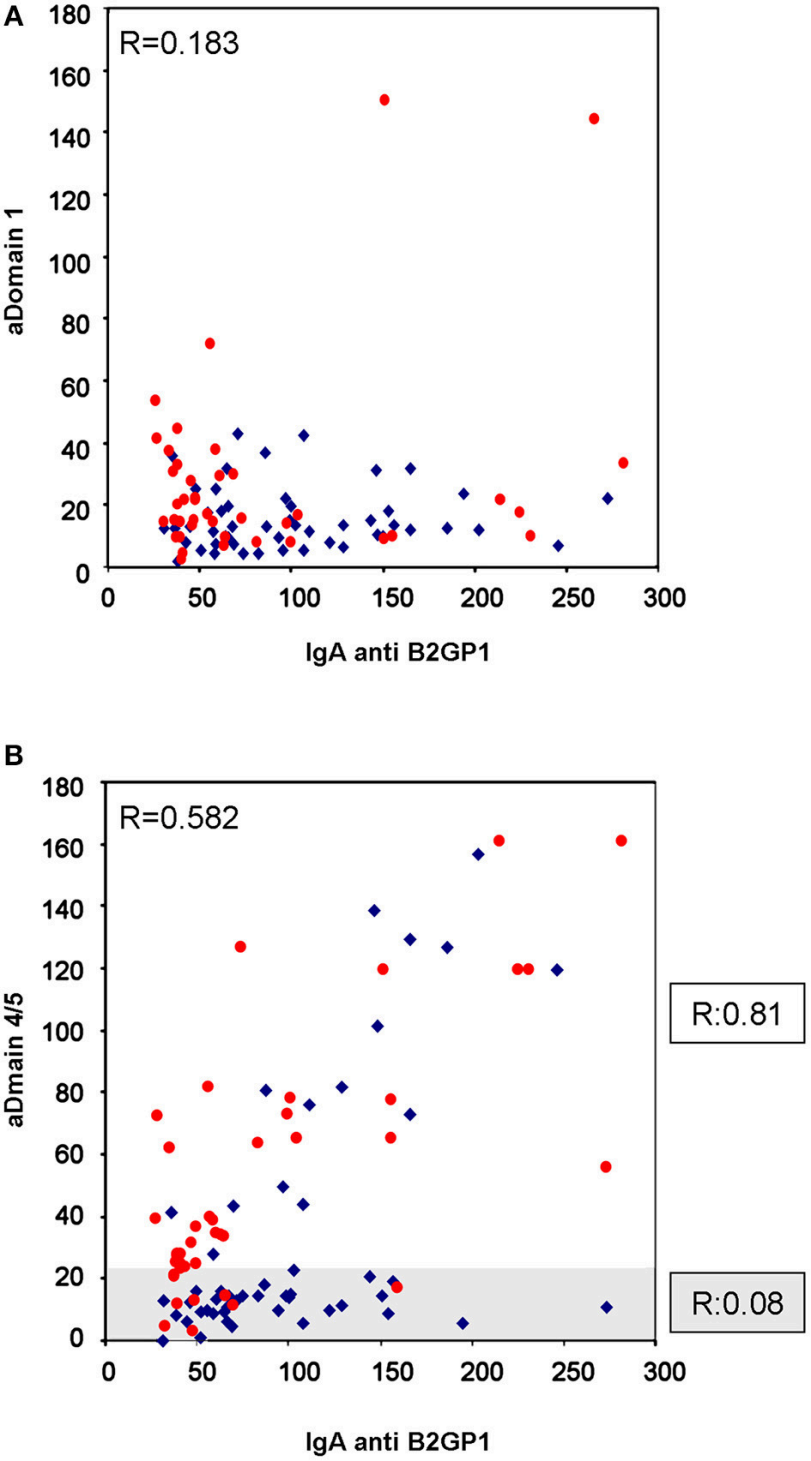
Correlation of IgA levels of anti-B2GP1 vs. anti-D1 and anti-D4/5. **(A)** Correlation of the levels of IgA anti-D1 and IgA anti-B2GP1 in the 93 patients with thrombotic events. No correlation should be found (*R* = 0.183; *p* = 0.079). **(B)** Correlation of the levels of IgA anti-D4/5 and IgA anti-B2GP1 in the same patients. In the overall study, moderate correlation was found (*R* = 0.582; *p* < 0.001). The graph has been dividend into two zones: the shaded zone includes the patients in whom no correlation is observed (*R* = 0.08). When only the patients of the non-shaded area are analyzed, a high correlation is observed (*R* = 0.81; *p* < 0.001). The patients with renal failure are in blue and those with normal kidney function in red.

A total of 47 patients (50.5%) were positive for Anti-D4/5 and 23 (25%) were positive for Anti-D1. No correlation was observed in antibody levels of IgA anti-B2GP1 and IgA anti-D1: *R* = 0.183 (95% CI: −0.022 to 0.373; *p* = 0.079; Figure 1A).

The levels of IgA anti-β2GP1 correlated significantly, although moderately, with the levels of Anti-D4/5: *R* = 0.582 (95% CI: 0.429–0.702; *p* < 0.001; Figure 1B).

When we closely examine the anti-B2GP1 vs. anti-D4/5 correlation graph Figure 1B, we can observe that there are two patterns of behavior in the patients' serum. In the first one—in 66 patients (clear area), there was a strong correlation (*R* = 0.81; 95% CI: 0.701–0.881; *p* < 0.001) and in the second pattern (subgroup of 27 patients, 29%) there was a practically null correlation (*R* = 0.08; shaded area in Figure 1B) To clarify, there was either a strong correlation in the patients or no correlation at all.

There were no significant differences in the proportion of anti-D1 positive among CKD and NKF patients (18 vs. 32%: *p* = 0.101). The proportion of patients positive for IgA anti-D4/5 was significantly lower in the CKD group (32 vs. 74%; *p* < 0.001).

Considering the 93 patients with thrombosis, no significant association was observed between the presence of the various types of thrombosis and the positivity of the IgA Anti-D1 or IgA Anti-D4/5 antibodies (Table 3).

**Table 3:** Clinical characteristics of patients with thrombotic events depending on the positivity of the presence of IgA autoantibodies to D1 or D4/5 of B2GP1.

	**aD1 negative**	**a D1-positive**		**aD4/5 negative**	**aD4/5-positive**	
**Characteristic**	**Number/mean**	**Number/mean**	***P*-value**	**Number/mean**	**Number/mean**	***P*-value**
Age (years)	57.1 ± 2.0	60.2 ± 3.3	0.434	56.8 ± 2.3	58.9 ± 2.5	0.540
Sex (men)	34 (46.6%)	9 (45%)	0.9	21 (46.7%)	22 (45.8%)	0.936
Deep venous thrombosis	45 (61.6%)	13 (65%)	0.784	29 (64.4%)	29 (60.4%)	0.689
Arterial thrombosis	12 (16.4%)	3 (15%)	0.877	8 (17.8%)	7 (14.6%)	0.676
Pulmonary embolism	12 (16.4%)	4 (20%)	0.709	7 (15.6%)	9 (18.8%)	0.683
Stroke	3 (4.1%)	3 (15%)	0.079	2 (4.4%)	4 (8.3%)	0.446
Myocardial infarction	8 (11%)	3 (15%)	0.62	5 (11.1%)	6 (12.5%)	0.836
Graft loss by thrombosis	9 (12.3%)	5 (25%)	0.160	9 (20%)	5 (10.4%)	0.197
Vascular access thrombosis	20 (27.4%)	2 (10%)	0.105	14 (31.1%)	8 (16.7%)	0.101

*No significant differences were observed*.

Furthermore, no significant differences were observed in patients with CKD with the presence of anti-D1 (*P* = 0.957) or anti-D4/5 and the occurrence of thrombotic events or loss of the graft by thrombosis (Table 4).

**Table 4:** Clinical characteristics of patients with thrombotic events associated with renal failure as a function of anti-D1 or D4/5 IgA autoantibodies positivity.

	**aD1 negative**	**a D1-positive**		**aD4/5 negative**	**aD4/5-positive**	
**Characteristic**	**Number/mean**	**Number/mean**	***p*-value**	**Number/mean**	**Number/mean**	***p*-value**
Age (years)	56.8 ± 2.4	55.9 ± 5.3	0.868	55.4 ± 2.7	59.3 ± 3.3	0.411
Sex (men)	18 (43.9%)	6 (66.7%)	0.216	17 (50%)	7 (43.8%)	0.680
Deep venous thrombosis	24 (58.5%)	7 (77.8%)	0.282	21 (61.8%)	10 (62.5%)	0.960
Arterial thrombosis	7 (17.1%)	2 (22.2%)	0.716	7 (20.6%)	2 (12.5%)	0.487
Pulmonary embolism	1 (2.4%)	0 (0%)	0.636	1 (2.9%)	0 (0%)	0.488
Stroke	1 (2.4%)	0 (0%)	0.636	1 (2.9%)	0 (0%)	0.488
Myocardial infarction	4 (9.8%)	1 (11.1%)	0.902	4 (11.8%)	1 (6.3%)	0.544
Graft loss by thrombosis	9 (22%)	5 (55.6%)	0.042	9 (26.5%)	5 (31.3%)	0.726
Vascular access thrombosis	19 (46.3%)	3 (33.3%)	0.478	14 (41.2%)	8 (50%)	0.558

*No significant differences were observed*.

### Analysis of Peptide Arrays

Of the 93 sera analyzed, 5 sera did not recognize any peptide in the peptide arrays (evaluated twice). The remaining sera recognized several peptides, although no common pattern was identified. The average number of peptides recognized by the sera of patients was 24.5 (28.6 in anti-β2GP1 asymptomatic carriers).

**Figure 2:**
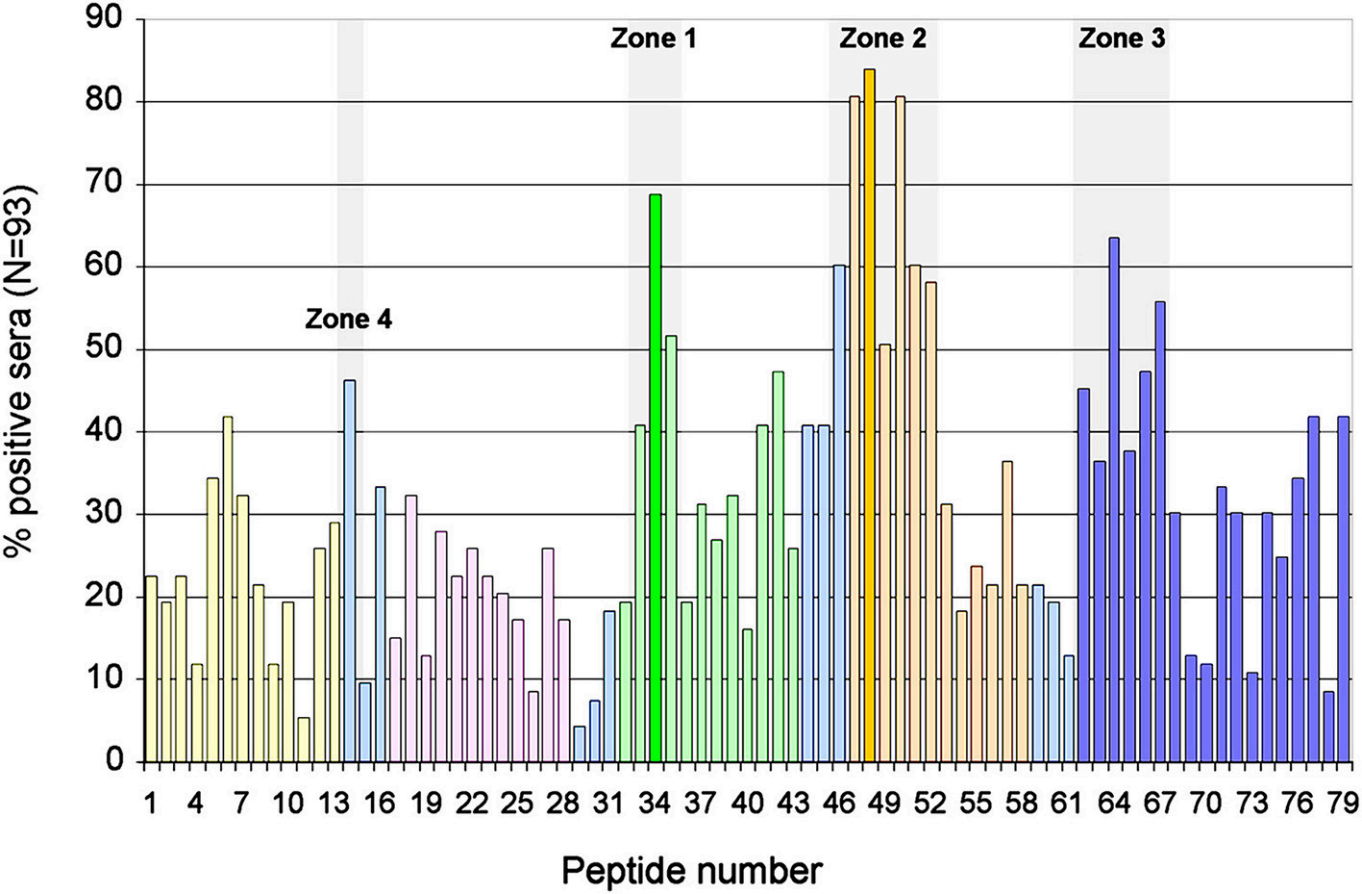
Identification of immunodominant regions of B2GP1. Description of the degree of antigenicity of the 79 peptides of B2GP1 evaluated in the 93 patients with thrombotic events. On the Y axis the percentage of sera that are positive for each of the peptides is indicated. Three peptides with high antigenicity corresponding to numbers 34 (P34; aa: 133–147), 48 (P48; aa 189–203), and 64 (P68; aa 253–267) were detected. Likewise, three areas of accumulation of peptides with positivity (gray overlap) located around these peptides can be observed: ZONE 1 (peptides 33–35) zone 2 (peptides 46–52), and zone 3 (peptides 62–67). The peptides corresponding to each domain are marked in: Domain 1, Yellow; Domain 2, Pink; Domain 3, Green; Domain 4, Orange; Domain 5, Dark Blue; Interlinking regions corresponding to two domains, cyan.

We assessed the degree of antigenicity of each peptide by the number of sera that recognized it (Figure 2). Highly antigenicity peptides were considered to be those that were recognized by more than 50% of the sera of patients with APS clinical signs. Three zones of the molecule were identified where there were peptides with high antigenicity: Zone 1 in domain 3, formed by the peptides 33–35 (P33-P35) and centered in P34 (aa140). Zone 2 in the domain 4 (P46-P52). As peptide P48 is the most recognized, it was considered to be the center of this zone, although the midpoint corresponds to aa 204. Zone 3 in domain 5 (P62-P67) in which peptide P64 was the most recognized of said zone (midpoint: aa264) (Figure 2).

Furthermore, a fourth zone located between domains 1 and 2 was also found: a peptide (P14) was recognized by 46% of the sera, very slightly below the cutoff of 50%, so that it was considered an antigenic-like zone to make comparisons. This was named zone 4. The three zones described were also recognized by the two groups of patients although region 4 was recognized better by the NKF group (not shown).

**Figure 3:**
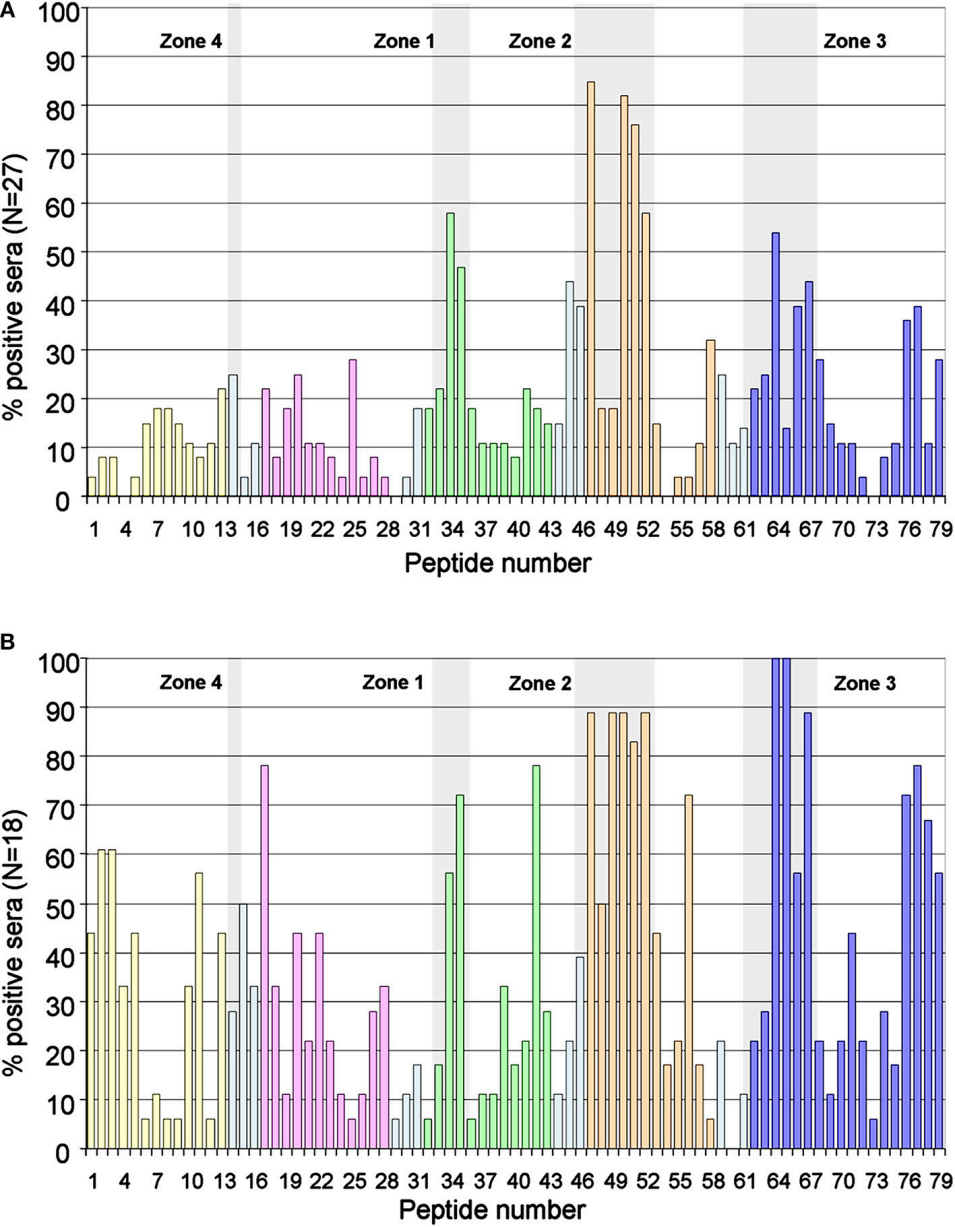
Immunodominant regions in the subgroup of patients D4/5 negative and in control group. **(A)** Description of the degree of antigenicity of the 79 peptides in the 27 patients with chronic renal disease and low values of anti-D4/5 antibodies. The four areas with accumulation of positivity are shaded in gray. **(B)** Antigenicity of the 79 peptides in the reference group of 18 asymptomatic carriers of IgA anti-β2GP1 (with other aPL negative). Response in the control group is more heterogeneous, however the three main antigenic zones can be identified. The peptides corresponding to each domain are marked in: Domain 1, Yellow; Domain 2, Pink; Domain 3, Green; Domain 4, Orange; Domain 5, Dark Blue; Interlinking regions corresponding to two domains, cyan.

Likewise, these zones were also recognized by the sera of the patients who were negative for the ELISA anti-D4/5 (Figure 3A). Of the 88 patients in whom peptides were recognized in domains 4 or 5, only 45 (51%) were positive in the anti-D4/5 ELISA test. Furthermore, only 12 (46%) of the 43 patients in whom some peptide were recognized in domain 1, only 12 were positive in the anti-D1 ELISA test.

The 18 asymptomatic IgA anti- β2GP1 carriers showed a polyclonal response, however the three zones described above can be clearly identified (Figure 3B).

### Localization of High Antigenicity Peptides in the Tertiary Structure of B2GP1

The tertiary structure of B2GP1 acquires a “fishhook” shape when open. If we observe the molecule from the right, it has an L shape and is called Face L. If we look at it from the other side, it has a J shape and is called Face J.

**Figure 4:**
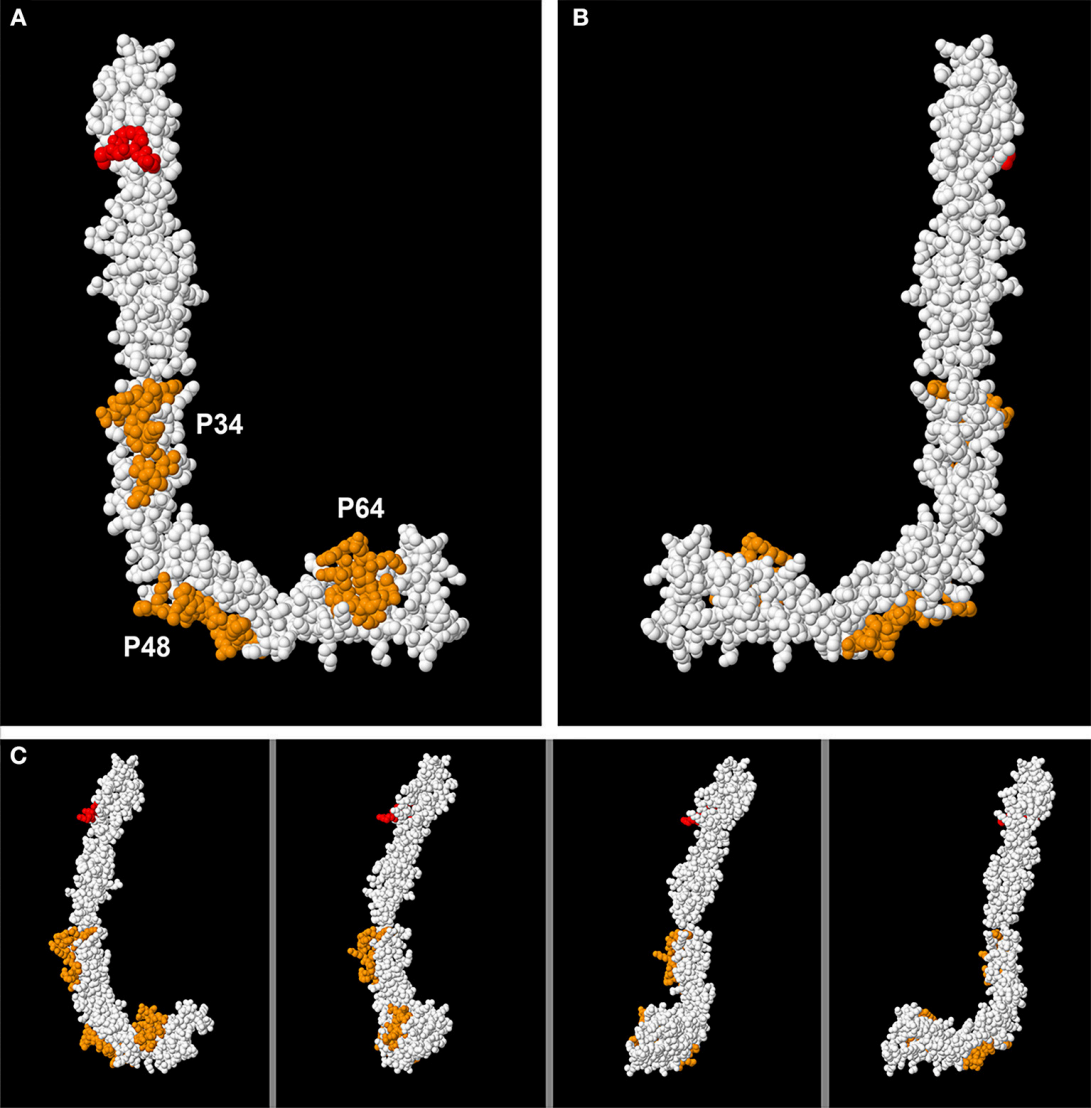
Visualization of the 3D structure of B2GP1 from the sides resembling the letter “L” and the letter “J.” **(A)** “L side” of B2GP1. Three peptides of high antigenicity (P34, P48, and P64) are indicated in orange. The R39-R43 epitope is indicated in red. **(B)** Visualization of the four previous epitopes in the “J side” of the molecule. Only a part of the P48 corresponding to the angle or “elbow” of the molecule is clearly observed. **(C)** Visualization of the epitopes of high antigenicity in 4 intermediate steps of the rotation process from the form in L to the form in J. In this process, it can be verified that the part of the P64 observed in the J face corresponds to a group of amino acids that protrude and that are really located in the L face.

Once the two faces are defined, if we mark the highly antigenicity peptides with colors, we can only see them on the L face, but not on the J Face (Figure 4A). Our three peptides (P34, P48, and P64) are shown in orange. The epitope on Domain 1 focused on the residues R39-G43 (23) is also marked, this time in red. However, when we turn the molecule and then observe face J, the peptides can no longer be visualized. Only one part of P48 can be observed, that which is located in the “elbow” of the molecule, part of it being visualized on Face J (Figure 4B). The intermediate steps of the rotation in Figure 4C from the layout in L to the layout in J can be visualized in this figure and in the Supplementary Video 1.

**Figure 5:**
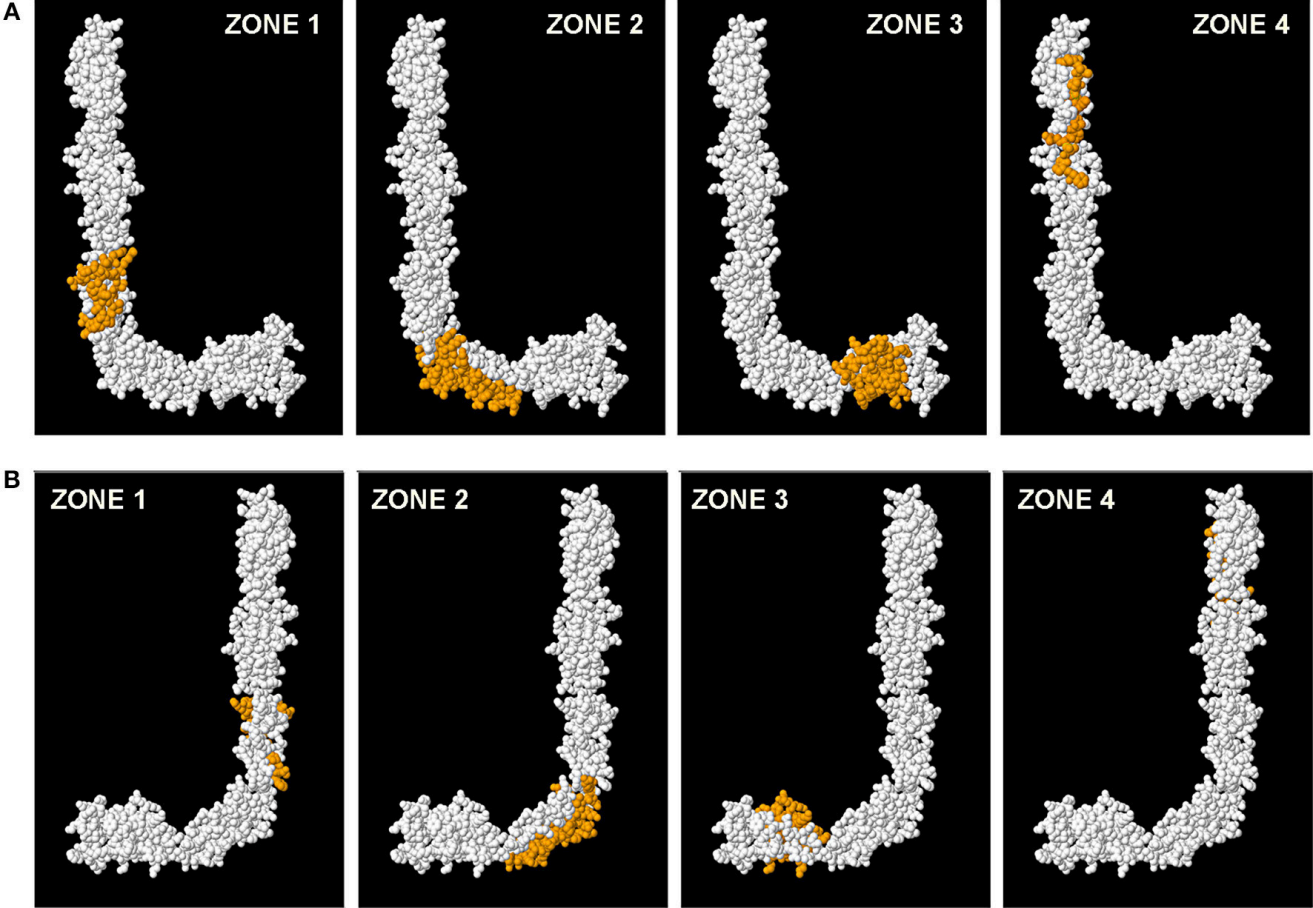
Localization of the four immunodominant zones in the structure of B2GP1. Visualization of the amino acids that correspond to each zone (marked in orange). **(A)** Observing the L face. **(B)** Observing the J face.

When we mark all the amino acids that correspond to the four zones (all the peptides of each zone) on the same model 3D of B2GP1, we can verify again that it is on face L where most of the amino acids recognized by the sera of the patients are located (Figure 5A). On face J (Figure 5B) only part of the amino acids corresponding to the angle of the molecule (zone 2). Zones 1, 3, and 4 can only be visualized from face L (see Supplementary Video 2).

## Discussion

In this work, we have demonstrated for the first time that the antibodies of isotype IgA from patients with thrombotic clinical signs consistent with APS who are only positive for the IgA against B2GP1 (negative for other isotypes) have affinity to specific zones of the B2GP1 molecule.

The group of patients studied include patients with normal kidney function and patients with end-stage kidney failure that represent the two clinical situations of the medical practice in which IgA anti-B2GP1 associated to thrombotic disease can have affinity to specific zones of the Target molecule be more frequently observed. This difference may be because the patients included are receiving very complete medical control, especially during the pre-transplant period during which they come to dialysis 3 times a week. This tight control facilitates the detection of the thrombosis in very early phases and results in immediate treatment before patients can evolve into pulmonary embolism.

### Antibodies Against ELISA Domains 1 and 4/5

The domain of B2GP1 identified as immunodominant in the response of anti-B2GP1 autoantibodies (IgG/IgM isotypes) was D1 (24). However, after having analyzed our Elisa-assay results and protein mapping analysis, we cannot consider that D1 behaves as immunodominant for anti-B2GP1 autoantibodies of IgA isotype. The determination of IgA anti-D1 antibodies does not seem to provide diagnostic advantages since they are less sensitive than the determination of the IgA antibodies against the complete molecule, as already described for the anti-B2GP1 IgG class (25).

Half of the patients were positive for the IgA anti-D4/5 antibodies, a level similar to that previously described by other authors in patients positive for the antibodies against the complete protein (11, 25). The correlation between the levels of the IgA anti-B2GP1 and IgA anti-D4/5 antibodies is strong, but only in 71% of the patients; it being almost null in the remaining 29% that do not recognized the D4/5.

These findings suggest that there should be other zones of the molecule involved in the recognition by the IgA of the patients and that detection of the class IgA and the anti-D1 and anti-D/45 antibodies cannot be used as the only substitute of the complete protein.

### Analysis of the Linear Epitopes

The recognition of the different peptides located in the peptide arrays by the IgA of the patients is wide-ranging and heterogeneous. Although there are some zones of the molecules that are more frequently recognized by part of the groups of patients, in the practice, each patient is different. These data suggest that the response of antibodies of the IgA isotype against the B2GP1 in our patients is polyclonal and directed against multiple zones of the molecule.

As it was not possible to localize profiles of patients, we decided to analyze the grade of recognition of each peptide by the sera of the patients. This strategy made it possible to identify both peptides that are highly recognized as zones in which there are peptides with a high grade of recognition.

It has been previously described that in addition to the classic cryptic epitope G40-R43 (26), there are other areas of B2GP1 molecule recognized by aPL (IgG and IgM isotypes) correlating with an increased risk for thrombosis. Blank et al., using pathogenic human monoclonal IgM antibodies anti-B2GPI (that can induce experimental APS) and a hexapeptide phage display library, identified three hexapeptides that react specifically with these antibodies (27).

These antibodies, called ILA-1 (28), ILA-3 (28), and H-3 (29), recognized the following peptides: Peptide ILA-1: 58-LKTPRV-63 (first and second domains of B2GP1). Peptide ILA-3: 208-KDKATF-213 (fourth domain of the B2GPI) and Peptide H-3: 133-TLRVYK-138 (this corresponds to the third domain) (27).

The Zone 2 of our mapping, the most frequently recognized by the sera of the patients, contains the sequence of the peptide ILA-3 in the P50-P53 peptides. An interesting fact in regards to future research is that the currently commercially available recombinant proteins formed by the 4/5 domains of B2GP1 and recommended for use in the ELISA technique do not contain the whole domain 4. In fact, they are truncated forms that lack the first 33 amino acids of domain 4. Thus, these proteins lack zone 2 and are not adequate to detect antibodies against this zone.

Zone 1 contains the hexapeptide recognized by H-3. The peptide 34, that having the highest grade of recognitions of the Zone 1, begins precisely with the six H3 amino acids.

The peptides corresponding to the cryptic epitope of the domain 1 (30) are only partially recognized. Very few sera react with the peptides that contain the motif R39-R43 (P8-P10). However, there is a moderate grade of recognition (46%) of the interlinking region between D1 and D2 that corresponds to the peptide containing the sequence of the exapeptide recognized by ILA-1 (P14). The different grade of recognition of the two parts of the epitope located in D1 could be explained because we are using linear peptides to identify antibodies that originally recognized a conformational epitope. With our current strategy, we only can detect antibodies that have high affinity for the linear part of the epitope and cannot detect the binding when dealing with the antibodies that recognize the conformational part.

Notably, the peptides recognized by ILA-1, ILA-3, and H3 are present in various microbial proteins. This fact suggests that molecular mimicry could be involved in the pathogenic production of these antibodies (31). The context in which the antigenic presentation of the microbial molecules is produced would direct that the response of the antibodies against them would be mediated by IgA if presented to the mucosal-associated immune system, or by IgM/IgG if presented in other organs and tissues of the immune system.

It is significant that the cryptic epitope D1, the sequences of amino acids recognized by the monoclonal antibodies ILA-1, ILA-3, and H3 and the antigenic zones described in this work cannot be visualized in face J and, nevertheless, all of them can be identified when we observed face L. This fact suggests that the two faces of the molecule probably have different behaviors from both functional and antigenic points of view.

The induction and stabilization of the “fishhook” conformation occurs after the binding of domain 5 to anionic structures (32). In the same way, the stabilization of the circular conformation of β2GP1 is due to the interaction between D1 and D5 of β2GP1 (6).

The epitopes suitable for binding antibodies (such as R39-R43) can only be exposed when the B2GP1 is arranged in the “fishhook” configuration. These epitopes would not be accessible in the circular and S forms (5, 33).

We do not know the localization of the zones we have described in the B2GP1 molecule when it acquires the circular form. As of the writing of this manuscript (September 2018), we have not found any data concerning the tridimensional structure of the circular conformation of B2GP1 in the Protein Data Bank (PDB) that could help us answer this question (34).

It could be hypothesized that the epitopes of Face L would all be cryptic and their accessibility would depend on the shape of the molecule, so that, as occurs with D1, they would lose their antigenicity when B2GP1 acquires the circular form. When the molecule takes on the circular form, the amino acids located in “Face L” could remain in the inside the ring after the union of domains 1 and 5, thus remaining inaccessible.

If, in contrast, they stay on the exterior circumference of the ring, the molecule would be strained because it is necessary for the exterior circumference to be larger than the interior one, producing a molecular reorganization with potential distortion and conformational changes of the molecule due to stretching that might distort the epitopes thus preventing a correct recognition by the antibodies.

It is known that in the preparation of the diagnostic systems to measure IgA anti-β2GP1 antibodies, the method used to prepare the antigen and how it is bound to the solid phase is a determining factor for the exposition of the epitopes recognized by the antibodies of the patients. The heterogeneity in the preparation various diagnostic systems can at least partly explain the variations in diagnostic performance and contributes to the often contradictory results in the literature on the relationship of APS clinical signs with the presence of the IgA isotype antibodies (35, 36).

If the localization of various zones with clinical interest is located in the same part of the molecule and within the same face, this can help to overcome the difficulties to implement sufficiently efficient diagnostic systems to detect the presence of the IgA anti-β2GP1 (35, 36). Furthermore, the determination of the spatial relationship between the epitopes recognized by the pathogenic antibodies could help to better understand the function of β2GP1 and APS pathogeny.

It is well-known that the presence of aPL is essential, but not sufficient, to trigger APS events. Additional triggering factors are probably needed to initiate the thrombogenic activity (second hit theory) (37). The hypothesis of the multiple cryptic epitopes could explain a possible second hit. The union of β2GP1 that circulates physiologically through the blood in circular form to anionic superstructures from microorganisms, or from injured endothelium in situations such as infections, surgery or traumatisms could result in a change in the conformation to the fishhook form and exposing the cryptic epitopes of the Face L. To further test the validity of this hypothesis, it is essential to have crystallographic data of the circular conformation, a task that will require assistance from the research community.

Identification by the antibodies of the IgA isotype of these zones of the molecule previously described in aPL of consensus and clearly related with the clinical signs of APS in animal models reinforces the findings in prospective studies of the strong association of the presence of these antibodies with the occurrence of APS events (11, 13, 38).

It also gives even more arguments to support that the IgA anti-β2GP1 antibodies are no longer the “Cinderella” (39) and that it is advisable to assess the possibility of including them in the APS classification criteria.

One of the limitations of our study is that we have used linear peptides that have allowed us to partially identify antigenic regions. In subsequent studies, a more detailed analysis needs to be performed, analyzing peptides having a smaller size and dissecting the sequences of amino acids with specific variations of the amino acid sequences to identify the zones of extensive affinity. The lack of access to crytallographic data of the circular and “S” shapes of β2GP1 has prevented us from making an analysis of the localization of the antigenic zones in these conformations.

In summary, we have described that the determination of the IgA against D1 and D4/5 separately does not improve the detection capacity of the systems using the complete protein to detect patients with IgA anti-B2GP1. Our results suggest that the anti-β2GP1 antibodies of the IgA isotype found in patients with clinical signs of thrombosis recognize zones of the molecule previously identified by the aPL monoclonals from patients with APS that are capable of inducing APS in animal models. The localization of these epitopes in face L of the molecule opens a new route to begin to understand the patholophysiology of the APS and to propose new alternatives and therapeutic targets.

## Ethics Statement

The study was approved by the Hospital 12 de Octubre Ethics Committee for Clinical Research (Reference Numbers CEIC PI13/405, CEIC 14/354 and CEIC 15/008). The patients were not asked to sign an informed consent because the Spanish legislation does not require it for observational studies without intervention in which genetic material is not used.

## Author Contributions

AS, JM-F, and MS designed the research. MS, JM-F, and JM were responsible for the patients' care and clinical data collection. JM-F, LN, and MS performed the determinations of anti-phospholipid antibodies. MS, JM-F and LN performed the determinations of IgA autoantibodies over the peptide-arrays. GN was responsible of the assays of Anti-D1 and Anti-D4/5 antibodies. AS and MS wrote the manuscript. AS conceived and directed the project. All authors discussed the results, contributed to the data interpretation, reviewed the manuscript, and agreed with the final version.

### Conflict of Interest Statement

GN is Director of Research and Development at INOVA Diagnostics. The remaining authors declare that the research was carried out in the absence of any commercial or financial relationships that could be construed as a potential conflict of interest.

## Supplementary Material

Supplementary Figure 1 Selection of patients and study disposition.

Supplementary Video 1 3-D motion of B2GP1 with the three most antigenic peptides (orange) and the “classical” D1 epitope (red).

Supplementary Video 2 3-D motion of B2GP1 with the three zones that accumulate peptides with the highest degree of antigenicity and the zone 4.

## Abbreviations

OR: odds ratio
anti-B2GP1: anti-βeta-2 glycoprotein-I antibodies
aCL: anti-cardiolipin antibodies
CIC: circulating immune-complexes
B2-CIC: circulating immune-complexes of IgG or IgM bounded to Beta-2 glycoprotein-I.
