# Supplementary figures and images for: The IgA Isotype of Anti-β2 Glycoprotein I Antibodies Recognizes Epitopes in Domains 3, 4, and 5 That Are Located in a Lateral Zone of the Molecule (L-Shaped)
Source: Front Immunol. 2019 May 7;10:1031. doi: 10.3389/fimmu.2019.01031 (PMC6515947; doi:10.3389/fimmu.2019.01031)

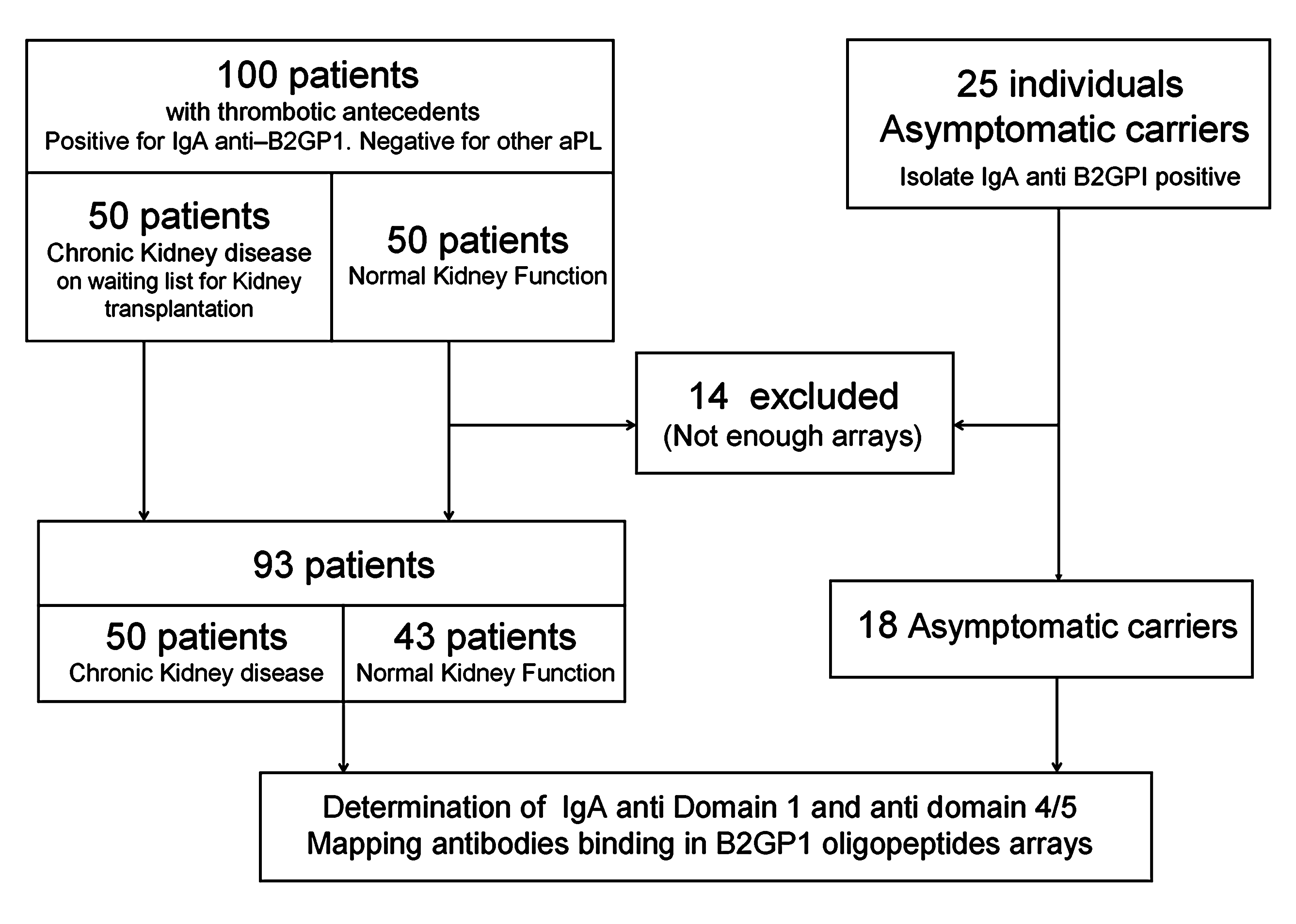

Supplement: Supplementary Figure 1 — Selection of patients and study disposition. [file Image_1.TIF]
